# 3D Graphene Foam by Chemical Vapor Deposition: Synthesis, Properties, and Energy-Related Applications

**DOI:** 10.3390/molecules27113634

**Published:** 2022-06-06

**Authors:** Cristina Antonela Banciu, Florin Nastase, Anca-Ionela Istrate, Lucia Monica Veca

**Affiliations:** 1 National Institute for Research and Development in Electrical Engineering ICPE-CA Bucharest, 313 Splaiul Unirii, 030138 Bucharest, Romania; cristina.banciu@icpe-ca.ro; 2 National Institute for Research and Development in Microtechnologies, IMT-Bucharest, 126 A Erou Iancu Nicolae, 077190 Voluntari, Romania; florin.nastase@imt.ro (F.N.); anca.istrate@imt.ro (A.-I.I.)

**Keywords:** 3D graphene foam, template-assisted chemical vapor deposition, supercapacitors, batteries, dye-sensitized solar cells, photodetectors

## Abstract

In this review, we highlight recent advancements in 3D graphene foam synthesis by template-assisted chemical vapor deposition, as well as their potential energy storage and conversion applications. This method offers good control of the number of graphene layers and porosity, as well as continuous connection of the graphene sheets. The review covers all the substrate types, catalysts, and precursors used to synthesize 3D graphene by the CVD method, as well as their most viable energy-related applications.

## 1. Introduction

Graphene, a two-dimensional single sheet of sp^2^ carbon atoms arranged in a hexagonal lattice, continues to draw extensive attention because of its peculiar properties. The theoretical specific surface area (SSA) of graphene is estimated to be about 2630 m^2^·g^−1^. Nevertheless, graphene tends to restack and form irreversible aggregates, which leads to a significant decrease in the surface area. To this end, different strategies for assembling 2D graphene in 3D architectures have been investigated, to preserve the large surface area of graphene [1,2,3]. A significant breakthrough occurred in 2011, when Chen reported an innovative approach to synthesizing template-assisted 3D graphene networks. The free-standing 3D graphene networks or graphene foams (GF) obtained by etching the metallic template were light and flexible, with an ultralow density of ~5 mg·cm^−3^, corresponding to the high porosity of ~99.7%; and a very high specific surface area, up to ~850 m^2^·g^−1^, corresponding to an average number of layers of ~3 [4]. The mechanical stability of the free-standing graphene enabled subsequent deposition of nickel oxide [5] or other inorganic/organic materials, to expand their functionality. Besides the larger surface area and high electrical conductivity, the elastic stretchability of GF is ~95% [4], which is much larger than the native stretchability of single-layer graphene (SLG ~6%) [6].

In general, these three-dimensional graphene networks are classified [7] both in relation to the (1) graphene engineering scale, and (2) connectivity of the graphene sheets. In the case of engineering scale, depending upon the dimensions of the pores, there are macroscopic 3D graphene (larger than 100 µm in one or more dimensions and comprising foams/sponges/aerogels monoliths, films, fibers, and millispheres) and microscopic 3D graphene (smaller than 100 µm in all dimensions and including powders of micrometer-scale or nanometer-scale 3D structures). In the case of graphene sheet connectivity, there are (1) joint 3D graphene architectures of individual graphene sheets interlinked via van der Waals forces; and (2) integrated 3D graphene networks, of continuously chemically linked carbon atoms. The second type offers a higher integrity, with superior conductivity and mechanical robustness.

The approaches employed to develop these 3D graphene networks include 3D template-assisted methods, and the self-assembly of graphene oxide (GO) into graphene sponges (GS). The first approach generates continuous and interconnected 3D graphene networks (graphene foams-GF), while the second creates an anisotropic structure, with the graphene sheets partially oriented or aligned almost parallel with each other. Another sponge-like 3D graphene structure is aerogel (GA) produced by sol-gel chemistry, which utilizes GO to form highly cross-linked graphene hydrogels (GH) [8].

Several reviews have been dedicated to 3D graphene networks, mostly prepared by assembling reduced graphene oxide (rGO) into 3D architectures, especially in connection with their potential applications as electrodes in supercapacitors, lithium-ion batteries (LIB), fuel cells, or for electromagnetic interface shielding [8,9,10,11,12].

The trends associated with various keyword combinations linked to the reviewed topic, over the 2011–2022 time period, are displayed in Table 1. A literature search on the Web of Science Core Collection identified a leading position for 3D graphene foam synthesis by chemical vapor deposition (CVD), with a peak in 2018, and the emerging development of their energy-related applications, many of which are related to supercapacitors and batteries. Similarly, the majority of the review articles were published in the field of graphene foam synthesis, with only two reviews in connection to CVD-grown graphene foams and energy storage and conversion. 

Herein, we have focused on the template-assisted 3D graphene networks synthesized using the CVD method, as it offers good control of the number of graphene layers, the continuous connection of the graphene sheets, and a high porosity. This is not an exhaustive presentation, but rather a concise review of the representative studies in the field, covering all the substrate types, catalysts, and precursors used to synthesize 3D graphene by the CVD method and their most viable energy-related applications, namely electrochemical energy storage (LIB and supercapacitors) and energy conversion (solar cells and photodetectors). For a comprehensive review of all types of 3D graphene-based structures, we suggest the Sun et al. review [7], which is an extensive review, covering all aspects of 3D graphene materials, including all methods used for 3D network production, with an accent on the GO/rGO self-assembly approach and properties, as well as their extended applications.

## 2. Synthesis and Processing Approach Using Chemical Vapor Deposition Method on Various 3D-Shaped Catalytic Templates

The template-assisted chemical vapor deposition (CVD) method is the most frequent method used to synthesize 3D interconnected graphene networks. This approach combines the 3D structure of the supported template with the special properties of the graphene, to form highly porous, light materials with excellent transport properties and mechanical strength.

**Scheme 1:**
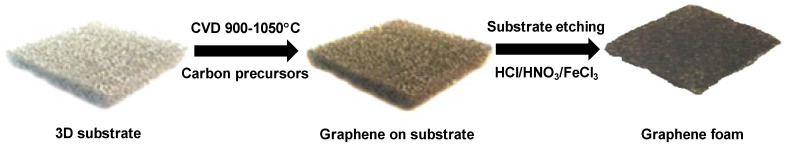
Schematic—presentation of the synthesis of 3D graphene networks by CVD.

Similarly to 2D graphene, the synthesis of the 3D graphene network, depicted in Scheme 1, follows the same path of carbon precursor decomposition on the growing substrates, in a stream of hydrogen–argon mixture. Characteristic of the 3D graphene network is the growing substrate, which functions both as the catalyst for the precursor’s decomposition and as a template for the final graphene architecture.

**Figure 1:**
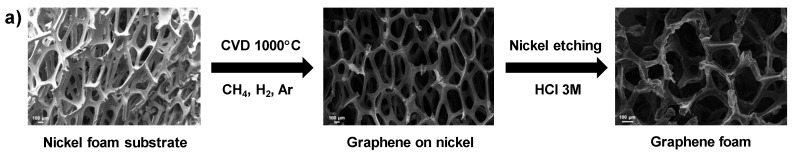

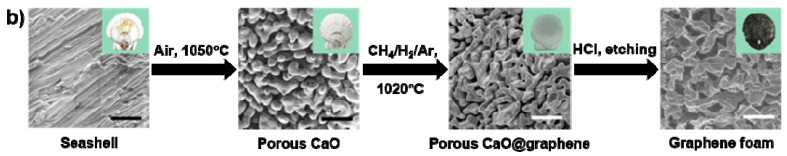
Synthesis of 3D graphene foam on (**a**) metallic and (**b**) seashell templates. First row (**a**): SEM images of Ni template, graphene-coated Ni after CVD growth, and free-standing graphene foam after removing the Ni template [13]. Second row (**b**): SEM images of pristine seashell microstructure, calcined seashell, graphene-coated calcined seashell after CVD growth, and 3D graphene foam after removal of the CaO template. All scale bars are 5 μm. Reprinted with permission from ref. [14]. Copyright the 2016 American Chemical Society.

While the growing substrate is either metallic or non-metallic, the carbon sources range from gaseous (e.g., methane, ethylene, acetylene) to liquid (ethanol, benzene, styrene, petroleum asphalt) and solid (polyvinyl-alcohol, camphor) phase. The CVD method involves these carbon sources that, at temperatures of 900–1050 °C and in a controlled atmosphere of hydrogen and argon, decompose in order to deposit the graphene layers on the substrate. An example of CVD-grown graphene on both metallic and non-metallic substrates is presented in Figure 1a,b, respectively [13,14]. The general procedure consists of template cleaning; hydrogenation to remove the oxide layer in the case of metallic substrate, or oxidation to generate the porous structure in the case of seashell; the CVD growth process; and template etching, to release the 3D graphene foam.

### 2.1. Catalytic Templates

Similarly to the well-established CVD routes based on planar substrates, the growing mechanism on porous scaffolds is surface-mediated self-limited growth, in the case of 3D graphene monolayers, or a segregation mechanism, in the case of 3D graphene multilayers. [11] While copper is the catalyst of choice to grow single-layer graphene, due to its low carbon solubility [15], nickel leads to mono- and multilayer graphene mixtures, due to the high carbon solubility. The critical characteristics of the template foam are the size, interconnectivity, and shape of the pores, as well as the type of substrate. Thus, to control the number of graphene layers and the porosity of the final graphene foams, different porous catalysts, and different synthesis conditions have been considered. To this end, the substrate skeletons range from foams [4,5,16,17,18] or films [19] to nanopowders [20,21,22], from metals to metal oxide [23], to metal salts [24] or seashells [14]. Regarding the distribution of the pores, they are either randomly distributed or well-ordered using 3D printing [25].

**Figure 2:**
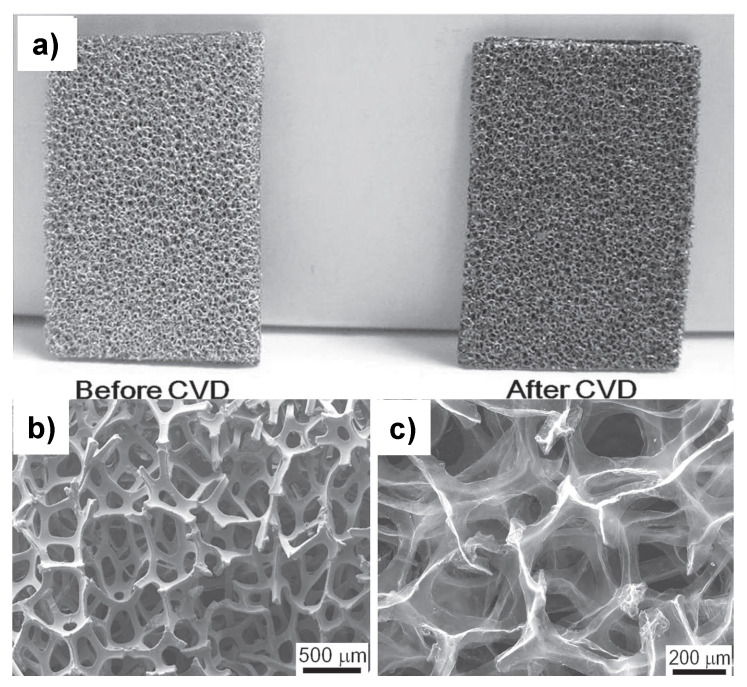
Photographs of (**a**) Ni foam before and after the growth of graphene, SEM images of (**b**) 3D graphene networks grown on Ni foam after CVD, and (**c**) 3D graphene networks after removal of Ni foam. Reprinted with permission from ref. [5]. Copyright 2011 WILEY-VCH Verlag GmbH & Co.

Due to its low price and availability in various sizes/pore densities and thicknesses, commercial nickel foam with a pore size of ~200 to 500 µm is the most frequently employed template [4,5,16,17,18]. Regardless of the employed precursor (methane [4] or ethanol [5]) the graphene is precipitated on the surface of the Ni foam at atmospheric pressure and temperatures of 1000 °C, forming a continuous, interconnected network that copies perfectly the shape of the Ni template (Figure 2a,b). Moreover, the graphene network retains its 3D structure after etching the supporting Ni scaffold (Figure 2c).

Aiming at improving the shape stability and thermal conductivity, a hierarchical graphene foam (HGF) macrostructure has been developed, starting from commercial Ni foam. The large and open pores of the Ni foam were filled with Ni- powder-PMMA slurry. Modified Ni foam templates later served as the template for the CVD growth of HGF. In this way, denser interconnected hollow graphene networks (HGN) were obtained [26].

**Figure 3:**
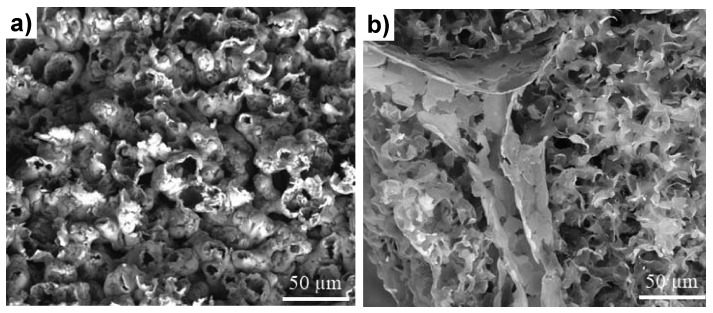
SEM images of the graphite foam samples growth on (**a**) nickel powder, (**b**) nickel powder-nickel foam hybrid. Reprinted with permission from ref. [22] with permission from Elsevier.

In a similar strategy, hybrid templates obtained from commercial reticulated nickel foam (400–500 µm pore size) infiltrated with nickel powder (44–88 µm and below 20 µm) were used to develop high surface area graphite foams [22]. The sintering temperature of the sacrificial catalytic templates was experimentally established for each type of powder, such as continuous, smooth, and interconnected networks between the nickel particles or nickel particles and nickel foam formed. As presented in Figure 3 the graphite foam replicated the structure of the metallic hybrid template. Sintering hybrid Ni foam and Ni powder reduced the pore size and increased the surface area of the graphite foam, to gravimetric and volumetric specific surface areas of 5.77 m^2^·g^−1^ and 0.52 mm^2^·m^−3^, respectively.

Further reducing the size of the starting Ni nanoparticles to ~500 nm allowed decreasing the pore size of the as-grown 3D graphene foam to around 1 µm [27].

Differently from the commercial Ni foam approach, a 3D porous Ni skeleton [28] was formed by reducing the NiCl_2_·6H_2_O in Ar/H_2_ atmosphere at 600 °C. This presents the advantage of developing macroscopic objects with relatively low porosity at atmospheric pressure, in a short time. Such 3D porous cross-linked Ni networks offer high mechanical and electrical properties, as well as low porosity.

In addition to the nickel nanopowders, 3D nickel nanowire foam was employed as a catalyst template to develop highly dense and interconnected 3D graphene [29]. The Ni nanowires synthesized from a Ni precursor solution under a magnetic field, in order to allow the formation of the nanowire, were physically assembled either by vacuum filtration or by pressing the Ni nanowire powder to form the 3D template for the subsequent graphene growth.

**Figure 4:**
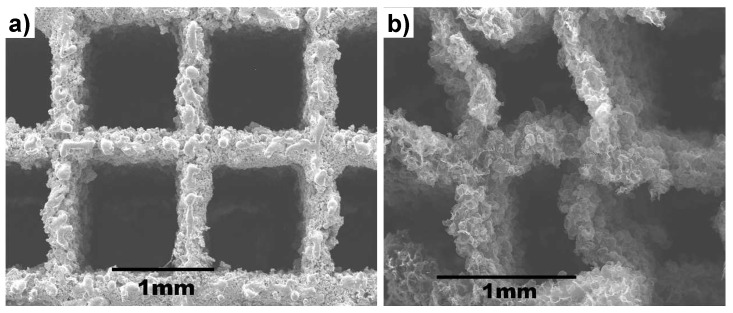
SEM image of the (**a**) as-synthesized 3D-printed Ni scaffold; (**b**) free-standing 3D graphene networks. Reprinted with permission from ref. [25]. Copyright 2015 The Royal Society of Chemistry.

In order to address the randomly arranged geometry and shape of the commercial Ni foam, the nickel template was 3D printed from Ni powder [25]. As depicted in Figure 4, this approach enabled the synthesis of well-defined pore structures/dimensions for both the metallic scaffold and free-standing GF.

Besides the classical Ni foams catalysts, 3D ultrathin graphite foam [30] was obtained, using an electrodeposited Ni scaffold as a template. First, the Ni catalyst was electrodeposited at high throwing power in a Ni sulfate bath on ordered polystyrene microspheres arrays, to generate a reverse-opal structure. This approach might open a new path for preparing graphene foams with a remarkable surface area and enhanced light harvesting.

Growing single-layer graphene at lower temperatures on copper catalyst was tested in a few reports, and low mechanical strength and discontinuous film formation at short deposition times were observed [31].

Three-dimensional-single (SLG) and few (FLG) graphene layers were grown on Cu and, respectively, Ni catalysts templets with a pore size smaller than 1 µm. The templates with a pore size of ~30 µm were obtained by sintering commercial powders to assemble and subsequently connect the metal particles in the 3D scaffold networks. The density and dimension of the pores were controlled by adjusting the sintering temperature and the diameter of the metallic particles. Unlike the commercial metal foams, which usually use compressed powders with particles of hundreds of microns in diameter in the presence of metal hydrides as blowing agents, this method employs particle sizes of tens of microns, without hydrides or compression, in order to prepare templates with a pore size one order of magnitude smaller than commercial foams [20].

**Figure 5:**
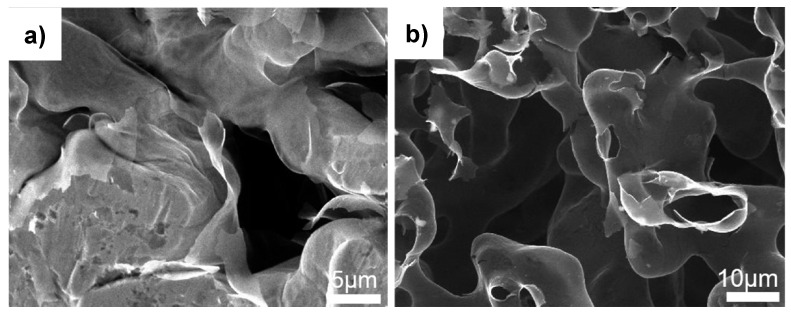
(**a**) SEM image and (**b**) the magnified SEM image of the 3D graphene network after evaporating copper template. Reprinted with permission from ref. [21]. Copyright 2017 American Chemical Society.

A 3D monolithic microporous copper template [21] with a pore size in the range of 10–20 µm obtained from copper powder allowed the synthesis of interconnected porous 3D graphene with a pore size 1–2 orders of magnitude smaller than those obtained on commercial nickel foam (Figure 5a). Furthermore, using a sublimation method to remove the Cu template, free-standing 3D graphene with high pore density and excellent mechanical properties was obtained without collapsing in the absence of the Cu skeleton (Figure 5b).

The smallest reported pore size of the Cu template (~1–4 µm) was prepared by partially removing zinc from commercial brass foil in a vacuum-dealloying method. Such a template enabled the development of porous graphene films with high electrical conductivity and high flexibility under tensile and bending strain [19].

In addition to a single metallic catalyst, Ni-Cu foam alloys were also employed to grow graphene foams with a controlled number of layers [18,32,33,34]. Starting from commercial nickel foam by melting and diffusion of the Cu film, they obtained Ni-Cu alloys that allowed them to control the number of graphene layers. While, in the case of polycrystalline Ni foam, few layers of graphene can be grown, in the case of Cu-Ni alloy, the grown graphene is mostly mono- and bilayer, with a dominant bilayer structure when the Cu content is 25%. This method offers the great advantage of using mono- and bilayer graphene on Ni–current collector electrodes for electrochemical energy storage [18]. On the other hand, the porous-nickel-plated Cu foil with a wide distribution of pore sizes (20 to 60 µm) allowed growing films with a low-density porous graphene network [34]. In this case, 3D graphene porous films were obtained by CVD, using porous Cu/Ni foils as catalyst substrate, obtained by electroplating the copper foil with nickel using the hydrogen bubble dynamic template method.

**Figure 6:**
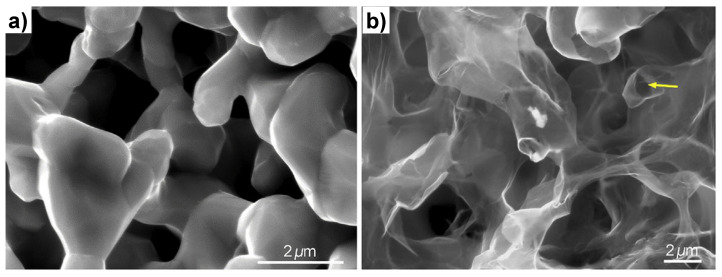
SEM images of (**a**) graphene-coated micron-porous Ni foam template; (**b**) micron-porous graphene foam after etching porous Ni template with the yellow arrow indicating the tubular shape of the pore. Reprinted from ref. [24] with permission from Elsevier.

Soft-templated metal frameworks [23] were developed based on the dextran property, to be an efficient template to form continuous monoliths of metal oxide foam. The pore size of such monoliths is an order of magnitude smaller than commercial nickel foam and offers the flexibility to prepare different types of catalysts, just by changing the metal salt. The metallic template catalysts generated by this method were copper, nickel, iron, and cobalt foams, which were subsequently used to grow macroporous graphene foams. Starting from Ni or Fe chlorides, porous metal templates were developed, in order to serve as a catalyst for the synthesis of micron-sized porous graphene foams [24]. The advantage of this precursor type comes from their reusability, as the Ni and Fe foams are chemically etched in HCl and, therefore, recovered from the waste to be used again in foam synthesis. As depicted in Figure 6a, the graphene film completely covers the Ni ligaments that form the template, and a graphene foam with micron-sized pores is preserved after etching the Ni skeleton (Figure 6b).

Although not the classical CVD-grown graphene, Lee et al. used a spin-coated PVA-NiCl_2_ film to prepare a 3D nano-foam with few-layer graphene [35]. After the thin films were dried in a vacuum for one day, the substrate was heated to a temperature of 1000 °C in an H_2_/Ar atmosphere at a pressure of 4 Torrs for 30 min, in order to carbonize PVA and form the 3D graphene nano-foam. The pore size estimated by TEM on an area of 25 µm^2^ was around 40–50 nm.

**Figure 7:**
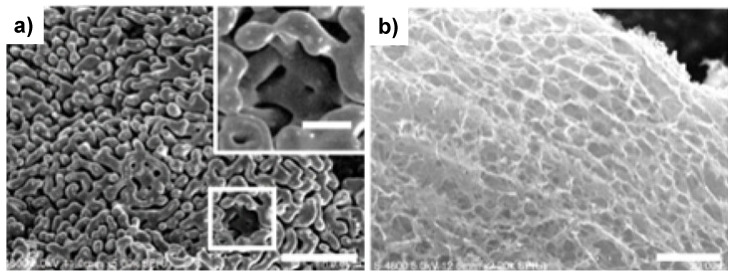
(**a**) SEM image of graphene layers deposited on the CaO surface; (**b**) SEM image of graphene foam with a layered structure. Scale bar for (**a**) is 5 μm, for inset in (**a**) is 2 μm, and for (**b**) is 10 μm. Reprinted with permission from ref. [14]. Copyright 2016 American Chemical Society.

A nonconventional catalyst to grow graphene foams was scallop substrates [14]. Structures with interconnected pores were obtained by thermal decomposition of CaCO_3_. The CaO that formed then served as the template for the CVD growth of graphene with high flexibility and electrical conductivity. The resulting CaO-graphene presented an average pore size of ∼300 nm. The SEM images of the graphene-CaO network presented in Figure 7 reveal the interconnected pores and layered structure of the graphene.

### 2.2. Precursors Employed in the CVD Process

Similar to 2D-grown graphene, a carbon source frequently used to synthesize 3D graphene is a mixture of methane and hydrogen [4,36,37], and depending on the ratio in the total gas flow and the growth time, the number of graphene layers can be controlled. Template-assisted CVD synthesis of high-quality 3D graphene foams was carried out in the presence of all types of precursors (gaseous, liquid, or solid) under different conditions (temperature, growing time, and gas flow). Chen et al. [4] found that the average number of graphene layers increased from 3 to 10 when increasing the methane concentration from 0.3 to 1.4 vol%. The second most commonly used gaseous carbon precursor is acetylene [38,39]. Different growing conditions yielded graphene (8 sccm flow rate, 1000 °C, ambient pressure, 4 min growth time) [38] or high-quality multilayer graphene (12 sccm flow rate, 900 °C, 15–60 min growth time) [39]. On the other hand, an ethylene precursor enabled graphene growth in 15 min at a temperature as low as 860 °C [40].

It is known that gaseous aliphatic hydrocarbons and hydrogen are dangerous and asphyxiating gases, extremely flammable, and can form explosive mixtures with air, which implies a high degree of safety precautions [41]. In order to overcome these safety issues, several studies reported cost-effective liquid carbon sources such as ethanol for growing single- and few-layer graphene on nickel foam [5,21,42,43,44,45,46,47,48,49]. In order to be used in CVD graphene synthesis, liquid precursors need to be transformed into gas before they reach the metallic catalyst. For instance, Cao et al. [5] used ethanol in the presence of argon to grow 3D graphene networks in 10 min at 1000 °C, with a cooling rate of 100 °C in the presence of an Ar–hydrogen mixture. Similarly, anhydrous ethanol was used to grow 3D graphene on nickel foam [44]. Three-dimensional graphene networks were also grown using anhydrous alcohol as the carbon source at 1000 °C for 10 min. However, unlike Huang et al. [44], a lower heating ramp under forming gas (N_2_:H_2_ = 95%:5%) was used [48]. In addition, a nickel-copper catalyst template and ethanol precursor were also used to grow 3D graphene networks at 1000 °C for 20 min under a hydrogen atmosphere [21].

At a somewhat lower growing temperature (600 °C) 3D graphene networks were grown on a Ni scaffold using benzene as a carbon liquid precursor [30]. In a standard CVD process, graphene was grown using styrene as a carbon source on a 3D printed nickel catalyst at 1000 °C for 1 h under an Ar–H_2_ gas mixture [25].

Several studies have been conducted with solid carbon precursors, such as petroleum asphalt and camphor [50,51]. Petroleum asphalt dispersed in toluene and coated on the nickel foam was thermally converted into 3D graphene networks at 950 °C and 1.5 × 10^3^ Pa under Ar/H_2_ gas mixture for 10 min [50]. As for the solid carbon precursor, camphor, it was introduced into the reactor at 200 °C near the gas inlet with a hydrogen flow of 200 sccm and a pressure of 20 mTorr for 15 min, to grow multilayers of 3D-graphene networks on a nickel foam catalyst [51]. Lee et al. [35], in an unconventional method starting from a mixture of PVA-NiCl_2_, containing both the carbon and catalyst precursors, developed 3D nano-foam. The PVA-NiCl_2_ spin-coated film on the SiO_2_/Si substrate was subsequently heated at a temperature of 1000 °C in an H_2_/Ar atmosphere at a pressure of 4 Torrs for 30 min, in order to carbonize PVA on the Ni catalyst and to form a 3D graphene nano-foam of few-layer graphene with high surface area and high conductivity [35].

### 2.3. Free-Standing 3D Graphene Foams

In the general process, free-standing graphene foam is obtained by etching the supporting substrate in strong acids, with or without the employment of the supporting polymer (poly (methyl methacrylate)-PMMA), but above room temperature and for a relatively long period of time.

Usually, free-standing graphene networks are released by etching the supporting metallic template in different acids or acids mixtures at temperatures ranging from 50 to 90 °C, and a relatively long period of time (from 3–4 h up to 48 h). HCl and a mixture of FeCl_3_/HCl in various concentrations (1 M to 3 M) are standard etching solutions for the metallic template [4,5,16,36,52,53,54]. However, other strong (HNO_3_ and Fe(NO_3_)_3_) [39,55,56,57], or mild acids [58] have been proven as efficient etching solutions. A combination of electrochemical and chemical etching processes [22] and sublimation [21] methods have also been used to effectively etch the metallic template.

**Figure 8:**
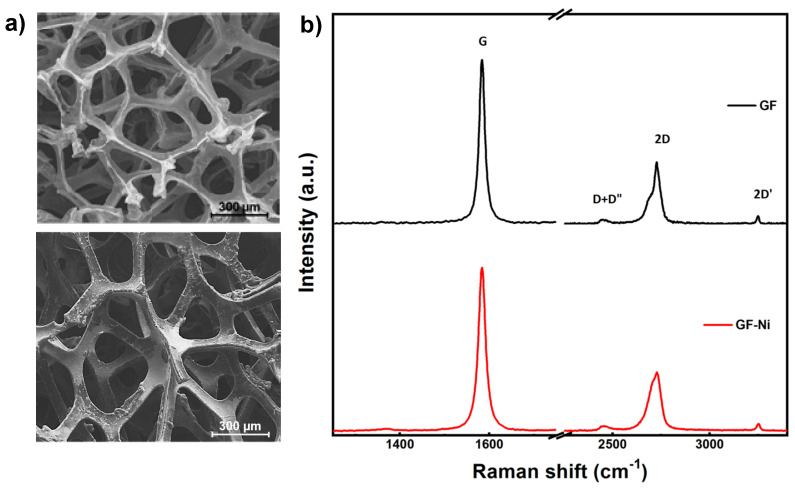
3D graphene structural and surface morphology characterizations: (**a**) SEM images for (bottom) as grown 3D graphene on nickel (GF-Ni) and (top) free-standing 3D-graphene (GF); (**b**) corresponding typical Raman spectra of (bottom) GF-Ni; (top) GF Reprinted from ref. [57] with permission from Elsevier B.V.

It is worth mentioning that the PMMA support layer used in the process of etching away the metallic substrate is a prerequisite only for thin graphene layers. When the graphene layers are increased above five, the metallic skeleton can be etched in the absence of a PMMA support layer [57]. As revealed in Figure 8a, even though the etching was carried out in the absence of the supporting PMMA polymer, the GF maintained its integrity after scaffold removal. A different behavior was observed when the thickness of the graphene foam was ~3 graphene layers [4]. In this case, the role of PMMA is to prevent the graphene network from collapsing.

Regardless of the etching solution, there is no evidence of graphene doping or defective site formation over the course of the etching process. Figure 8b shows the Raman spectrum of free-standing 3D graphene foam after etching in HNO_3_, in comparison to the as-grown graphene on Ni (GF-Ni) and shows no shifts in the G position or the appearance of structural disorder. Similarly, according to different studies, the structural characteristics of as-grown graphene persist after etching in solutions of HCl, and HCl/FeCl_3_ [4,5,55].

## 3. Properties of 3D Graphene Foams

Since their first synthesis in 2011, 3D graphene networks have revealed unique properties, such as high specific surface area (SSA), good electrical conductivity, and mechanical strength. Table 2 summarizes the reported values as a function of the number of graphene layers and density.

Divergent data can be seen, with the graphene macrostructures exhibiting densities ranging from 5 mg·cm^−3^ [4] up to 100 mg·cm^−3^ [21], and electrical conductivity varying from 10 to 1600 S·cm^−1^ [21]. With respect to the SSA, the highest reported value is ~1590 m^2^·g^−1^ [61], which is lower than other carbon nanomaterials (activated carbon or activated graphene oxide), which exceeds 3000 m^2^·g^−1^, or metal-organic frameworks (MOFs) with an SSA up to 10,000 m^2^·g^−1^ [62]. Nevertheless, large pores and low density come at the cost of device performance drops, as nanomaterials with low packing density will generate empty space in the electrode, which will be filled by the electrolyte and will increase the weight of the device without adding capacitance. 

## 4. Energy-Related Applications

Synthesized 3D graphene on 3D templates by the CVD method demonstrated not only their flexibility and high conductivity, but also a high mechanical strength. Owing to these properties, the material has shown increased interest for a plethora of applications, including energy storage and conversion. The main efforts were devoted to the field of energy storage, namely supercapacitors and batteries, with limited but evolving interest in solar cells and photodetection.

Expected to ensure efficient ion and electron transport properties, 3D graphene has been directly employed as a current collector in energy storage devices. Moreover, owing to the foam’s appropriate hierarchical structure for supporting electro-active materials, such as metals, metal oxides, sulfides, or hydroxides, GF-based hybrids have been successfully developed and exploited as electrode materials in energy storage devices. The main advantage of these hybrids comes from their capacity to function in the absence of binding agents, which are detrimental to the electrical conductivity and electron transport needed for a high cyclability and rate capability. 

### 4.1. Energy Storage-Supercapacitors

Supercapacitors have a high power capability, a long cycle lifetime, and fast charge and discharge rates. Supercapacitors have the potential to complement or eventually replace rechargeable batteries for some energy storage applications, but they suffer from a lower energy density (~5–8 Whkg^−1^) compared to rechargeable lithium-ion batteries (120–200 Whkg^−1^).

According to their charge storage mechanism, there are two types of supercapacitors: electrochemical double-layer capacitors (EDLC), and pseudo-capacitors. An EDLC stores charge electrostatically, with the charged ion located at the surface of the electrode material.

The energy storage principle in a supercapacitor device is dominated by the electrostatic charge accumulation on an electrode–electrolyte interface, commonly known as electrical double-layer capacitance.

Carbon-based materials are mostly used as electrode materials for EDLC, due to their high specific surface area, good electrical conductivity, and large pore size distribution; while pseudo-capacitors are made from redox-active materials, such as transition metal oxides or hydroxides and conductive polymers.

In hybrid supercapacitors, the next-generation high-performance supercapacitor devices, the charge storage is obtained by combining two types of charge storage mechanisms, capacitive non-Faradaic (EDLC) and capacitive Faradaic (pseudocapacitive), to improve the overall energy storage capacitance of the supercapacitor [63]. 

It should be stressed that it is not a facile task to directly and unequivocal compare the performances of energy storage devices, unless all parameters are taken into account. Thus, in the absence of complete data, in the following sections, we present a summary of the reported characteristics of 3D-graphene-based electrodes, or the final supercapacitor when this has been reported. Table 3 summarizes the main performance features of several electrode/supercapacitor configurations in which 3D graphene was used. 

**Figure 9:**
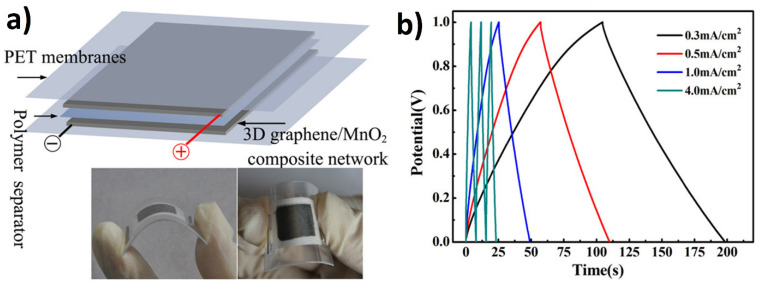
(**a**) Schematic of the flexible supercapacitors consisting of two symmetrical graphene/MnO_2_ composite electrodes, a polymer separator, and two PET membranes. The two digital photographs show the flexible supercapacitors when bent; (**b**) Galvanostatic charging/discharging curves of the flexible supercapacitor device at different current densities. Reprinted with permission from ref. [64]. Copyright 2012 American Chemical Society.

He and collaborators, [64], reported the development of a free-standing, lightweight (0.75 mg∙cm^−2^), ultrathin (<200 µm), highly conductive (55 S∙cm^−1^), and flexible 3D graphene network, loaded with MnO_2_ by electrodeposition, as the electrodes of a flexible supercapacitor; presenting a stable performance at different current densities (Figure 9).

The large surface area of the 3D graphene allowed a large MnO_2_ mass loading (~92.9% of the entire electrode), leading to a high areal capacitance of 1.42 F·cm^−2^ at a scan rate of 2 mVs^−1^. The maximum specific capacitance was 130 Fg^−1^, calculated for the entire electrode. The highest specific capacitance was 465 Fg^−1^ for the sample with a mass loading of 0.1 mg·cm^−2^, showing a decrease in the specific capacitances with increasing MnO_2_ loading. However, the specific capacitance of the entire electrode increased with the increase of the content of active material and reached a maximum for an active material of 55–75% at scan rates of 2, 10, 50, and 100 mVs^−1^. At the lowest scan rate of 2 mVs^−1^, the specific capacitance of the entire electrode remained as high as 130 Fg^−1^, even when the mass of active material was up to 92.9%, indicating that the active material has an excellent capability to contribute to the capacitance of the entire electrode, owing to the lightweight nature and high electrochemical performance of the graphene/MnO_2_ composite electrode. There was no significant difference between the CV curves with and without bending, suggesting the high flexibility of the 3D graphene/MnO_2_ composite network-based symmetrical supercapacitor; the MnO_2_ loading, in this case, was 0.4 mg·cm^−2^. The flexible supercapacitor exhibited an energy density of 6.8 Whkg^−1^ at a power density of 62 Wkg^−1^ for a 1 V window voltage. It also preserved 55% of its energy density as the power density was increased to 2500 Wkg^−1^. The capacitance of this supercapacitor decreased from 29.8 Fg^−1^ to 27.8 Fg^−1^ after 500 cycles, and the Coulombic efficiency remained above 93%. After 5000 cycles, it retained a high specific capacitance of 24.2 Fg^−1^, about 82% of the Coulombic efficiency. The specific capacitance retained 92% of its initial value, even after 200 bending actions, with a bending angle of 90°, indicating its excellent mechanical and flexible properties.

In 2014, Zhang et al. [46] developed a graphene-based three-dimensional hierarchical sandwich-type architecture (graphene/carbon nanotubes/Mn_2_O_3_) and tested its performance as a supercapacitor. The sponge-like 3D graphene/CNT was obtained by the CVD method, employing ethanol as a carbon source, and Mn_2_O_3_ was obtained using an electrodeposition process. Graphene was grown on commercial Ni foam, followed by immersion in NiCl_2_, to prepare Ni nanoparticles as the catalyst for the CVD growth of the CNT. After etching the Ni foam, the Mn_2_O_3_ was electrodeposited on the sponge-like architecture of graphene/CNT. The discharge areal capacitance of the graphene/CNT/Mn_2_O_3_ electrode at 100, 250, 400, and 500 mAg^−1^ was 370, 320, 301, and 280.9 Fg^−1^, with a 72% weight of Mn_2_O_3_. The composite electrode also had a good Columbic efficiency; even after ~1000 cycles of charge/discharge, the curves remained symmetrical, with a specific capacitance of ~180 Fg^−1^.

In 2015, Zang and collaborators [36] fabricated flower-like NiCo_2_O_4_ on 3D graphene foam (GF) and tested it as electrodes in supercapacitors. The 3D GF was prepared by CVD, with a subsequent electro-deposition of flower-like NiCo_2_O_4_. A maximum specific capacitance of 1402 Fg^−1^ was achieved at a current density of 1 Ag^−1^. The NiCo_2_O_4_/GF nanohybrid-based supercapacitors exhibited long-cycle stability, with 76.6% retention of its specific capacitance after 5000 cycles at a current density of 5 Ag^−1^. The CV differed from those of electrical double-layer capacitors, which usually produce a CV curve close to an ideal rectangular shape. In this case, the CV consisted of pairs of sharp redox peaks, suggesting the contribution of pseudo-capacitance to the total capacitance of the electrode. The redox peaks were prominent, even at 100 mV/s, which indicates a fast redox process and diffusion of ions in solution.

Wang [65] used a 3Dgraphene/Ni_3_S_2_ composite as electrodes in supercapacitors. Whereby, 3D graphene/Ni_3_S_2_ composites were obtained in a two-step process involving CVD growth graphene on Ni foam and hydrothermal synthesis of Ni_3_S_2_. The composite electrodes exhibited a specific capacitance of 11.529 F∙cm^−2^ at 2 mA∙cm^−2^, that is 2611.9 Fg^−1^ at 5 mVs^−1^, and retention of 88.97% capacitance after 1000 charge/discharge cycles at 20 mA∙cm^−2^. Ni_3_S_2_ was deposited on the GF foam obtained after etching the Ni substrate. Ni_3_S_2_ presented low crystallinity and the best electrochemical performance was observed for the sample synthesized for 6 h. The composite structures obtained demonstrated that the active materials were equally distributed on the surface of the Ni_3_S_2_, which was beneficial for the electrochemical reaction of the composites and improved their electrochemical performance. Capacitance of 3DG/Ni_3_S_2_-6h composites at various current densities: 11.529 F·cm^−2^ at 2 mA·cm^−2^; 10.424 F·cm^−2^ at 5 mA·cm^−2^; 9.448 F·cm^−2^ at 10 mA·cm^−2^; 9.12 F·cm^−2^ at 20 mA·cm^−2^.

**Figure 10:**
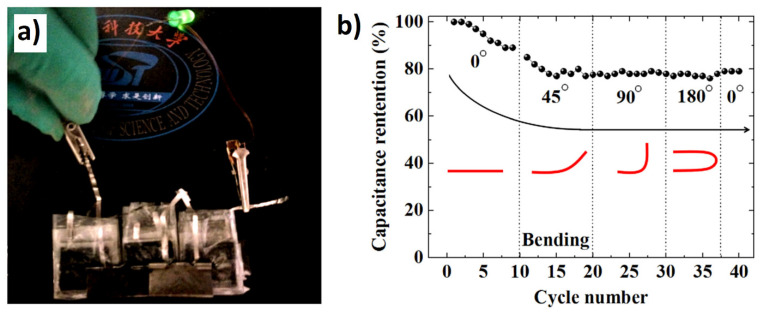
(**a**) A demonstration of six FSCs connected in series lighting a green LED bulb; (**b**) Capacitance retention of the FSC based on MnO_2_/MGF/NF with a loading time of 12 h, with a dynamic bending process. Reprinted from ref. [37] with permission from Elsevier B.V.

In 2017, Liu [37] developed a 3D foam-like MnO_2_ film/multilayer graphene film/Ni foam (MnO_2_/MGF/NF) for flexible supercapacitors (Figure 10).

The area (volumetric) capacitance reached 714 mF·cm^−2^ (14.28 F·cm^−3^) with good cycling stability of 98% after 5000 cycles. The maximum volumetric energy (power) density reached 0.634 mWh·cm^−3^ (126 mW·cm^−3^), at a power density of 6.22 mW·cm^−3^.

Usman [66] developed 3D vertically aligned Ag nanoplates on nickel foam-graphene (NFG) using electrodeposition with sonication as an efficient supercapacitor. The composite exhibited a specific capacitance of 900 Fg^−1^ at an applied current density of 0.5 Ag^−1^, it also showed long-term cyclic stability after 5000 cycles, and the Columbic efficiency was almost 99% of the initial value. During the electrodeposition, the whole cell was placed on an ultrasonication apparatus with a power of 50 W. The presence of the ultrasounds favored the formation of the vertically aligned Ag NPs. The specific capacitance retention was 60% after 5000 cycles, showing reasonable cyclic stability. The energy density was 35 Whkg^−1^ at a power density of 0.91 kWkg^−1^, and 26 Whkg^−1^ at a power density of 12.4 kWkg^−1^.

Cheng fabricated ternary composite electrode-supercapacitors from nitrogen-doped graphene foam/CNT/MnO_2_ via CVD and a facile hydrothermal method [67]. The CNT was grown on the NGF–nitrogen-doped GF (after etching the nickel substrate) using a CVD method, followed by hydrothermal deposition of MnO_2_ nanoflakes. NGF can provide more active sites for electron storage and also enhances the binding energy with ions in the electrolyte. Larger binding energy leads to a larger number of ions being accommodated on the electrode surface and the enhancement of the binding energy improves the capacitance. The electrochemical properties of the flexible ternary electrode (MnO_2_ mass loading of 70%) exhibited an areal capacitance of 3.03 F·cm^−2^. At the scan rate of 2 mVs^−1^, the specific capacitance of the entire electrode remained as high as 284 Fg^−1^, even when the mass loading of MnO_2_ was up to 70%, indicating that the active material in the hybrid structure had an excellent capability to contribute to the capacitance of the entire electrode. Additionally, as a key parameter of supercapacitor electrode performance, the cycling ability of an NGF/CNT/MnO_2_ 70% electrode was studied using CV tests at a scan rate of 50 mVs^−1^ for 15,000 cycles. The capacitance showed a 51.6% increase after 10,000 cycles, then remained stable from 10,000 to 15,000.

Madito and collaborators, [18], grew graphene on both Ni and Ni-Cu foams as electrodes in supercapacitors. Ni-Cu foam alloys were prepared using 3D polycrystalline nickel foam. The active material on the Ni-Cu graphene foam current collector displayed a higher electrochemical performance, in terms of the calculated specific capacitance, compared to Ni and Ni-graphene foam current collectors. The active material on the Ni-25%Cu graphene foam current collector showed a specific capacitance of 289.7 Fg^−1^ at a current density of 1 Ag^−1^, and at a high current density of 5 Ag^−1^, the specific capacitance remained as high as 106.7 Fg^−1^. An AC (activated carbon as active electrode material) with Ni-15%Cu graphene foam current collector showed a specific capacitance of 257.8 Fg^−1^ at a current density of 1 Ag^−1^, and at 5 Ag^−1^ the specific capacitance remained as high as 81.6 Fg^−1^. Ni-foam loaded with AC showed a specific capacitance of 57 Fg^−1^ at a current density of 1 Ag^−1^.

Li fabricated 3DG/ZnO composites, in which ZnO nanorods were hydrothermally grown on 3DG structures [47]. Graphene was obtained by CVD on Ni foam, using ethanol as a carbon source, as the larger structural polarity of ethanol allows growing graphene at low temperature. The best results were obtained for the 4 h hydrothermal growth, when the coverage of the graphene surface with the ZnO was completed/sufficient and the diameter of the nanorods was 125 nm. Composites of 3DG/ZnO, were electrochemically tested and showed high specific capacitance as supercapacitor electrodes and a high electrochemical stability. The composite presented a specific capacitance of 554.23 Fg^−1^ at 5 mVs^−1^, as well as good reversible charge/discharge ability (~94.4% retained after 2300 cycles). The high performance of the prepared supercapacitors was assigned to the superiority of the porous structure, due to the assistance of mass transfer of electrolyte ions. It had an excellent long cycle life over the entire process and excellent cycle stability, with a high degree of reversibility in repetitive charge/discharge.

Drieschner and collaborators [20] synthesized a 3D GF with a small pore size, as small as 1 µm, by tuning the particle size of the metallic nanoparticles. The GF obtained from these foams had a volumetric electric double layer capacitance of 165 mF∙cm^−3^, and a specific capacitance of 100 Fg^−1^, when the number of graphene layers was decreased to a single layer, which gives them potential as supercapacitors. The small pore size increases the stability of the GFs in comparison to those grown on commercial metallic foams. In addition, 3D SLG- and FLG-based GFs with controllable pore size provided a highly-specific volumetric and surface electric double layer capacitance when the as-produced foams were used as electrodes in aqueous electrolytes. A GF grown on templates with a pore size down to 1 µm has a more compact and dense structure, resulting in a higher EASA per volume. The highest volumetric capacitance was obtained for Cu(Mg) 1 µm grown at 850 °C, around 165 mF·cm^−3^, and specific capacitance of 100 Fg^−1^, and capacitance per area of 59 mF cm^2^. The corresponding energy and power densities were 11.2 Whkg^−1^ and 10.1 kWkg^−1^, respectively. The charge/discharge measurements indicated the characteristic fingerprint of an ideal capacitive interface for the GF electrodes. Testing carried out over 10,000 cycles at a volumetric current of 4 mA∙cm^−3^, resulted in a charge time of 3 s; typical for supercapacitor applications. The tests revealed no significant degradation of the electrode performance, with a total capacitance drop below 1%.

**Figure 11:**
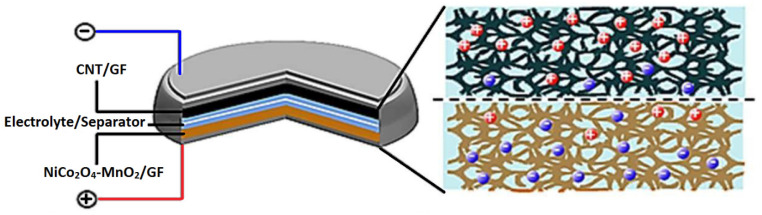
Schematic of the designed asymmetric coin supercapacitor. Reprinted with permission from ref. [68]. Copyright 2017 The Royal Society of Chemistry.

Garakani and collaborators [68] fabricated heterogeneous NiCo_2_O_4_–MnO_2_ arrays consisting of a mesoporous NiCo_2_O_4_ nanowire core and a crosslinked MnO_2_ nano-sheet shell grown on a graphene foam (GF), for ultra-high performance supercapacitors. The developed composite electrodes had a specific gravimetric capacity of 2577 Fg^−1^ to 1 Ag^−1^ and a retention capacity of 94.3% after 5000 cycles. An asymmetric supercapacitor assembled with NiCo_2_O_4_–MnO_2_/GF and CNT/GF composites as positive and negative electrodes, respectively, provided a maximum specific energy of 55.1 Whkg^−1^ at a specific power of 187.5 Wkg^−1^ (Figure 11).

Liu and collaborators [69] developed Ni(OH)_2_/G-NF/Ni(OH)_2_ as the electrode in a supercapacitor, which showed an specific capacity of 991 Cg^−1^ at a current density of 1 Ag^−1^, good rate capability of 70.7% at 20 Ag^−1^, and with a retaining capacity of 95.4% after 5000 cycles.

**Figure 12:**
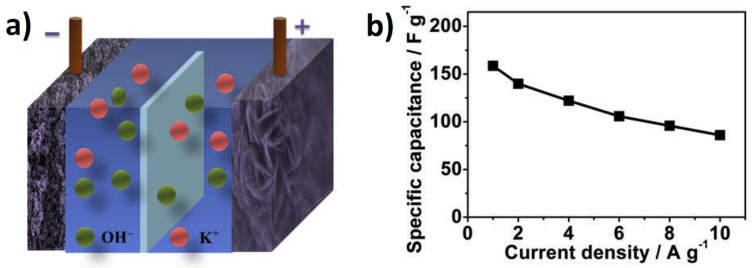
(**a**) Schematic illustration of the assembled hybrid supercapacitor. (**b**) Specific capacitance at different current densities. Reprinted from ref. [69] with permission from Elsevier Ltd.

Moreover, their hybrid supercapacitor, assembled from Ni(OH)_2_/G-NG/Ni(OH)_2_ as the positive electrode and AC/NF as a negative electrode with 1 M KOH as the electrolyte and cellulose paper as the separator, delivered a specific energy of 49.5 Whkg^−1^ at a power density of 750 Wkg^−1^ (Figure 12). The hybrid supercapacitor also exhibited a power density of 7500 Whkg^−1^ at the specific density of 26.9 Wkg^−1^ with an excellent lifespan, with 89.3% retention after 10,000 cycles at 10 Ag^−1^.

**Figure 13:**
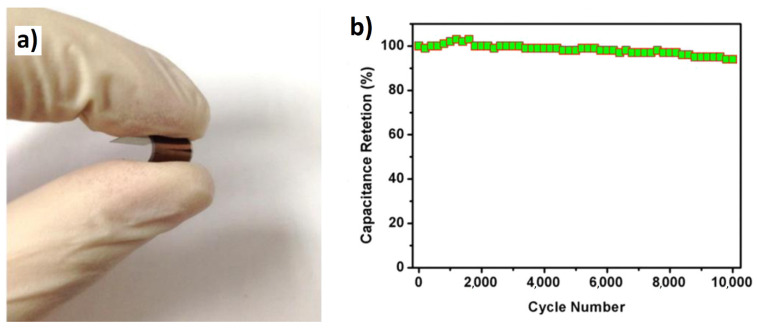
(**a**) Digital picture of the solid-state device (size ~1 cm × 0.8 cm) under folded conditions; (**b**) Cycling stability of the device at a current density of 2 Ag^−1^. Reprinted from ref. [70] with permission from Elsevier Ltd.

Qin reported the synthesis of a hierarchical nanoporous graphene (hnp-G) with a high specific surface area (1160 m^2^·g^−1^) and its performance as a binder-free electrode for flexible solid-state supercapacitors (Figure 13) [70].

A symmetric supercapacitor PET/Au/hnp-G/PVA solid gel electrolyte/hnp-G/Au/PET allowed continuous and short electron/ion diffusion pathway in the entire device, yielding a capacitance of 38.2 F·cm^−3^ and excellent rate performance. The reported energy density was 2.65 mWh·cm^−3^ and the power density was 20.8 W·cm^−3^, with 94% retention after 10,000 cycles.

In 2011, Cao [5] fabricated electrochemically deposited NiO on a 3D graphene network and tested it as the electrode in a supercapacitor, showing a specific capacitance of ~816 Fg^−1^ at a scan rate of 5 mVs^−1^, and stable cycling performance after 2000 cycles. The direct synthesis of graphene on the Ni current collector offered an effective electrical contact for fast charge transfer from the active material to the current collector through the graphene network, which covered the Ni foam and held the active material (NiO).

Xiao used the nickel foam coated with graphene to deposit Ni(OH)_2_ on the surface using a hydrothermal method [38]. The Ni(OH)_2_/graphene structure was investigated using cyclic voltammetry, to demonstrate their use as electrodes in pseudocapacitors. These structures developed a high specific capacitance (1440 Fg^−1^ at a charge/discharge current density of 10 Ag^−1^) and a good cycling performance (~100% capacitance retention over 2000 cycles in 1 M NaOH electrolyte and current density of 20 Ag^−1^); thus, presenting applicability for energy storage.

Hsia used ultrathin graphite grown on a templated electrodeposited Ni layer to fabricate close-packed hollow 3D graphitic bicontinuous foam [30]. Electrodes were synthesized for supercapacitors, starting from porous structures of Ni scaffold-electrochemically deposited on PS and used as a catalyst to grow “ultrathin graphite” by CVD at 600 °C and with a benzene precursor. Employing methane and high temperature (1000 °C) for the deposition was avoided, as the Ni reverse opal electrodeposited structure would collapse at temperatures above 600 °C. The advantage of the method comes from the good ion transport in the porous structure and high surface area, as both sides of the graphene spheres are used. Based on the measured capacitances and the theoretical specific surface areas per volume, an approximate capacitance per real surface area of ~20–30 µF·cm^−2^ was derived. The capacitance was 2–8 times higher than the typical double-layer capacitance of carbon-based electrodes [71]. The highest areal capacitance of 1 mF·cm^−2^ was achieved for a 10-layer sample of 0.5 µm diameter spheres. The highest measured volumetric capacitance of 2.7 F·cm^−3^ was achieved on a five-layer sample with 0.5 µm diameter spheres. The areal capacitance showed an increase when increasing the number of PS layers, with no difference between spheres of 0.5 and 1 µm. On the other hand, in the case of volumetric capacitance, this was higher when spheres of 1 µm were used in comparison with 0.5 µm, but there was no significant contribution from the number of layers. The maximum calculated volumetric energy and power densities for the five-layer, 1 µm diameter PS in a three-electrode configuration was 382 mJ·cm^−3^ and 3.2 W·cm^−3^, which is considered comparable to other novel 3D graphitic materials.

### 4.2. Energy Storage-Batteries

The advantages of using 3D graphene in battery structures are derived from their high conductivity, which ensures efficient ion and electron transport. Moreover, 3D graphene is light and flexible compared to other metal electrodes, such as Ni, yet mechanically strong and with an electrical conductivity suitable to being directly employed as a current collector, without a binder and conducting agent. Moreover, the functionality of the 3D graphene foams was extended by their decoration with different transition metal oxides, sulfides, or semiconductors of various dimensions and morphologies. 

The 3D architecture of the graphene and its unique properties allowed the flexibility to develop both anode and cathode materials with high surface area and implicitly high active material loading.

Table 4 summarizes the performance of several types of batteries where 3D graphene was used in various configurations.

#### 4.2.1. Lithium-Ion Batteries (LIB)

Lithium-Ion Batteries (LIB) are the main power source in the portable electronics market. Recently, use of LIB has expanded to hybrid electric vehicles, electric vehicles, and even grid-scale energy storage. A commercial LIB anode is typically graphite, but this has a theoretical specific capacity of ~372 mAhg^−1^, which is considered to be low to be employed as a large-scale LIB for high power consumption. The required characteristics of electrodes for LIB include high capacity, excellent cycling performance, and rate capability. Various nanostructures, such as nanoparticles, nanowires, nanotubes, nanowalls, porous nanosheets, and hollow nanoparticles, have been developed for this purpose. The advantages of nanostructures as LIB electrodes are their short transport path and an increased electrode/electrolyte contact area, which is beneficial for high current rate performance. In such structures, the strain could be significantly reduced during the lithiation and delithiation process, preserving the integrity of the electrode and leading to a more stable cycle performance.

In 2014, Huang [44] grew MoO_2_ nanoparticles on 3D graphene derived from ethanol by CVD, with Ni foam as the scaffold. These binder-free electrodes presented a high specific capacity and rate performance and could be successfully used as anodes in LIB. They had high reversible capacities of 975.4 mAhg^−1^ and 537.3 mAhg^−1^ at the current densities of 50 and 1000 mAg^−1^, respectively. The electrode also showed an increased capacity, from 763.8 mAhg^−1^ to 986.9 mAhg^−1^ after 150 discharge and charge cycles at a current density of 200 mAg^−1^. It was observed that over 100 cycles, the MoO_2_–3DG nanocomposites maintained a stable discharge capacity of ~520 mAhg^−1^. The MoO_2_–3DG nanocomposites were able to deliver reversible discharge capacities of 975.4, 899.1, 800.3, 716.9, and 537.3 mAhg^−1^ at current densities of 50, 100, 200, 400, and 1000 mAg^−1^, respectively. Furthermore, the capacity was restored to a stable stage when the current density returned to 50 mAhg^−1^. This implies that the material is highly stable and reversible.

Lim and collaborators [48] developed a WS_2_-3D graphene nano-architecture as anode material for LIB applications. The 3D-graphene network provided mechanical support for the WS_2_ nanostructure, as well as enhanced conductivity and ionic kinetics in comparison to commercial WS_2_ powder. The nanocomposite exhibited reversible capacities of 927 and 416 mAhg^−1^ at current densities of 100 and 1500 mAg^−1^, respectively. It was also observed that at a current density of 200 mAg^−1^, WS_2_-3D graphene nanocomposites can retain a capacity of 748 mAhg^−1^ after 500 cycles, with a Coulombic efficiency (CE) higher than 98%; indicating outstanding cycling stability. To ensure a good surface coverage with the WS_2_, the graphene network was treated in air plasma for different periods of time (30, 60, and 90 s), with the best electrochemical performances being obtained for the specimen treated for 60 s; being able to deliver specific capacities of 927, 815, 630, 500, and 416 mAhg^−1^ at current densities of 100, 200, 500, 1000, and 1500 mAg^−1^. It was also observed that the specific capacity returned to 820 mAhg^−1^ when the current density was adjusted from 1500 to 100 mAg^−1^.

Deng used MnO_2_ nanoflakes as an LIB anode, assembled on CVD-grown GF via the facile hydrothermal process [55]. It was tested without any binder, conductive additives, or other current collectors and showed a capacity of 1200 mAhg^−1^ at a current density of 500 mAg^−1^ and a capacity higher than 500 mAhg^−1^ at a current density of 5 Ag^−1^, as well as a long cycle life of up to 300 cycles. The capacity of the electrode (binder-free) decreased in the initial 50 cycles, but increased gradually to 1200 mAhg^−1^ after 300 cycles. The discharge capacity reached 1200, 1080, 899, and 600 mAhg^−1^ at a current density of 0.5, 1, 2, and 5 Ag^−1^, respectively. The capacity reverted to 1200 mAhg^−1^ when the current density returned to 0.5 Ag^−1^.

Güneş and collaborators [72] obtained a GF/SiNWs composite with a Si mass loading above 0.3 mg·cm^−2^, which was tested as an anode material in a LIB. The 3D porous architecture of the GF/SiNW exhibited high gravimetric and areal capacities and long cyclic life. The performance was further improved after alumina coating the SiNW using the ALD technique. In the first cycle at 200 mAg^−1^ current density, both GF/SiNWs composites and their ALD coated counterparts showed similar gravimetric discharge capacities of 2423 and 2949 mAhg^−1^. When increasing the number of cycles, and at a current density of 500 mAg^−1^, the GF/SiNW composite showed faster decay compared to the ALD-coated specimen. The ALD coated sample showed 83% capacity retention (2074 mAhg^−1^), while the composite without alumina retained only 76% after 20 cycles. The discharge capacity decreased to 1125 mAhg^−1^ and 875 mAhg^−1^ after 50 cycles for the ALD-coated and uncoated specimens, respectively. ALD coating induced better cycling stability. ALD coated GF/SiNW showed stable and reversible capacities at high rates, with 850 mAhg^−1^ and 550 mAhg^−1^ at current densities of 2000 and 4000 mAg^−1^, respectively.

Wang fabricated 3D NiMoO_4_ nanowire arrays (NWAs) grown directly on the surface of macroporous graphene foam (GF–after Ni etching) and tested this as an anode material for LIBs [56]. The NiMoO_4_ (NWAs)/3DGF composites showed a reversible specific capacity of 1088.02 mAhg^−1^ at a current density of 200 mAg^−1^ and 867.86 mAhg^−1^ after 150 cycles (79.77% retention of the second cycle) and excellent rate capability. The Coulombic efficiency increased from 84.92% in the first cycle to 98% in the second cycle, and then remained above 99% during subsequent cycles. The rate performance of the NiMoO_4_/3DGF was tested at current densities ranging from 200 to 3200 mAg^−1^. All specific reversible capacity results were moderately decreased with increasing current density. Even at a current density of 3200 mAg^−1^, a capacity of 770 mAhg^−1^ was obtained. More importantly, after high-rate discharge-charge cycles, a discharge capacity of about 915 mAhg^−1^ could be resumed and maintained for another 10 cycles, without obvious decay when the current density was 200 mAg^−1^ again. The superior rate capability was attributed to the direct coupling of the active material onto the current collector; thereby greatly enhancing the charge transfer properties.

Tang and collaborators [16], aiming to increase the electronic conductivity of an olivine LiFePO_4_ anode in a LIB, prepared a 3D graphene/LiFePO_4_ composite to be tested as anode material for LIB, presenting a good rate performance of 109 mAhg^−1^ at 10C. The charge/discharge capacities were measured in a classical coin cell. A specific discharge capacity of 158 mAhg^−1^ was obtained at a rate of 0.2C; a value that decreased to 150, 144, and 135 mAhg^−1^ at 1, 2, and 5C. Even at a high rate of 10C, the specific discharge capacity was still 109 mAhg^−1^. Cells discharged to 69% of their initial capacity at a rate of 10C, while LiFePO_4_ alone (without graphene) fell to only 26%, at the same discharge rate.

Luo developed a bicontinuous mesoporous nanostructure Fe_3_O_4_ on 3D graphene foam using ALD and directly used it as a LIB anode [73]. The electrode exhibited high reversible capacity and a fast charging–discharging capability. A high capacity of 785 mAhg^−1^ was achieved at a 1C rate and showed no decay up to 500 cycles. A rate up to 60C was demonstrated, providing a fast discharge potential. It demonstrated a capacity of 780 mAhg^−1^ at 1C and 350 mAhg^−1^ at 10C up to 500 cycles.

#### 4.2.2. Lithium-Sulphur Batteries (LSB)

Lithium-Sulphur Batteries (LSB) are considered next-generation batteries, owing to their high theoretical gravimetric (~2600 Whkg^−1^) and volumetric (~2800 WhL^−1^) energy density. The theoretical specific capacity of sulphur ~1675 mAhg^−1^ is much higher than that of conventional materials of lithium transition metal oxides. Moreover, the nominal energy density of Li-S batteries is 2500 Whkg^−1^ of cell weight, while the energy density of the mainstream Li-ion batteries is ~150 Whkg^−1^.

**Figure 14:**
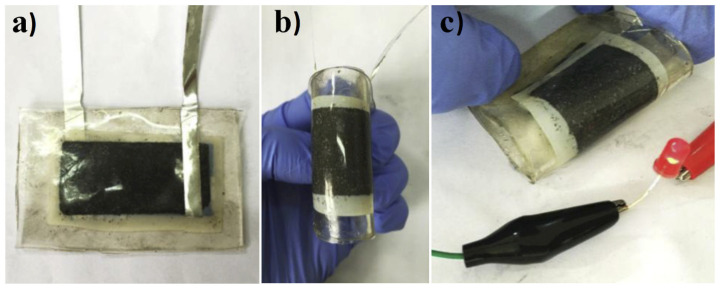
(**a**,**b**) Photographs of the flat and bent battery encapsulated by PDMS, showing the good flexibility of 3DLG. (**c**) Lighting a red LED device under bending. Reprinted from ref. [74] with permission from Elsevier.

He developed a 3D Li_2_S/graphene hierarchical architecture (3DLG) synthesized by Li_2_S infiltration into a 3D graphene network prepared by CVD on Ni-foam (Figure 14) [74].

The material was employed as a free-standing binder-free cathode without metallic current collectors or conducting additives. The 3DLG exhibited a high discharge capacity of 894.7 mAhg^−1^ at 0.1C, a high-capacity retention of 87.7% after 300 cycles at 0.2C, and a high-rate capacity up to 4C, when it reached 598.6 mAhg^−1^. The calculated capacity retentions relative to the initial cycle after 100, 200, and 300 cycles were 86.5%, 84.4%, and 82.4%. The cathode exhibited an excellent rate performance, with reversible specific capacities of 894.7, 853.2, 801.3, 745.8, 683.1, and 598.6 mAhg^−1^ at 0.1, 0.2, 0.5, 1, 2, and 4C. Additionally, the 3DLG restored the majority of the original capacity (886.5 mAhg^−1^) as the C-rate was set back to 0.1C, indicating that the 3DLG maintained its structure under different current rates.

Xi developed a lightweight three-dimensional cathode for Li-S batteries [75]. After etching, the Ni sulphur was loaded on the framework using a solution infiltration method. The battery using sulphur-FLG foam cathode showed good electrochemical stability and high-rate discharge capacity retention for up to 400 discharge/charge cycles at a high current density of 3200 mAg^−1^. After 400 cycles the capacity decay was ~0.064% per cycle, and the average Columbic efficiency was ~96.2%.

Shi and collaborators grew 3D graphene with mesopores, onto a metal oxide template [76]. The porous graphene framework (PGFs) obtained after etching the template offered a highly mesoporous structure with interconnected graphene nanocages. These graphene networks, obtained after removing the template, were employed as conductive scaffolds to host sulphur for lithium-ion storage. The performance of the PGF-S cathodes was tested at a potential range of 1.7–2.8 V and demonstrated excellent cycling stability. An initial discharge capacity of 1187 mAhg^−1^, corresponding to a very high sulphur utilization of 71%, was achieved for PGF-S cathodes at a current rate of 1C. The capacity decreased to 1063 mAhg^−1^ after one cycle. The capacity remained at 852 mAhg^−1^ after 500 cycles, which corresponds to a capacity fading of only 20% and a decay rate of 0.04% per cycle, starting from the second cycle. In the case of DTG-S (double-layer templated graphene) cathodes, the initial discharge capacity was 1086 mAhg^−1^, which slightly dropped to 1030 mAhg^−1^ in the second cycle, preserving the capacity of 634 mAhg^−1^ after 500 cycles, giving a capacity decay of 38% and fading rate of 0.08% per cycle. The rate performance of the PGF-S cathode showed a decrease in capacity when increasing the current density from 0.1 to 10C. The capacity of 609 mAhg^−1^ was preserved at a current density as high as 5C, indicating the superior rate capability of the tested cathode. Moreover, after harsh cycling conditions at a rate of 10C, the capacity was as high as 980 mAhg^−1^ when the current density was returned to 0.1C.

Free-standing 3D graphene obtained on metallic salts templates was coated with PMMA and loaded with sulphur in the presence of carbon black, to develop a low-density binder-free and metal-free 3D-graphene current collector for Li-S batteries (3D-MPGF-Sx) [24]. The loading with sulphur spanned from x = 2.5 to 7 and 13 mg∙cm^−2^, which corresponds to/equals 63, 82, and 90 wt.% of sulphur content, with the lower S loading electrode exhibiting a higher reversible capacity than the higher S-loading electrodes. The initial discharge and charge capacities of the electrode with loading x = 2.5 mg∙cm^−2^ sulphur, reached 1352 and 1269 mAhg^−1^, leading to a Coulombic efficiency of 93.9%; on the other hand, increasing the loading to x = 7 and x = 13 yielded lower discharge and charge capacities (loading 7 mg∙cm^−2^ sulphur-1027 and 884 mAhg^−1^ with a Coulombic efficiency of 86% and loading 13 mg∙cm^−2^ sulphur-791 and 637 mAhg^−1^ with 80% efficiency). The electrode with the sulphur loading x = 2.5 mg∙cm^−2^ had the highest initial discharge capacity of 1187 mAhg^−1^ at 0.1C, while the discharge capacity of the 50th cycle decayed to 618 mAhg^−1^, corresponding to a retention of 52%. The Columbic efficiency was ~96.5%. In this study, even after 50 cycles, the capacities of all 3D-MPGF-Sx electrodes remained at 400 mAhg^−1^. The excellent high capacities of the electrodes were mainly attributed to the low density and conductive networks of 3D-MPGF.

#### 4.2.3. Zn-Air Batteries

Rechargeable metal–air batteries are a promising technology, due to their high theoretical energy densities. 

**Figure 15:**
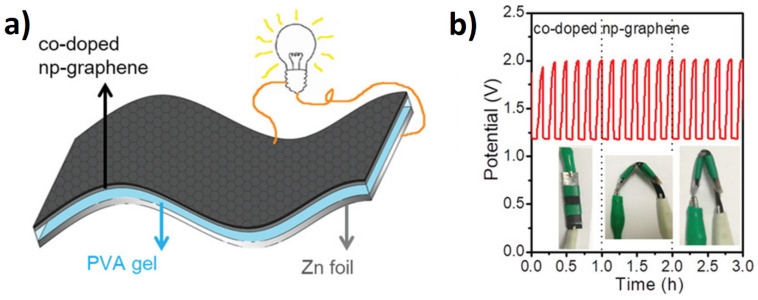
(**a**) Schematic diagram of the co-doped np-graphene-based all-solid-state Zn-air battery; (**b**) Discharge/charge curves under different bending states. Reprinted with permission from ref. [77]. Copyright 2019 WILEY-VCH Verlag GmbH & Co.

Qiu developed a flexible Zn-air battery by assembling free-standing np-graphene (~30 µm thick) as the air cathode, a Zn foil (0.1 mm thick) as the anode, and a polymer gel as the electrolyte (Figure 15) [77].

Graphene, N-doped graphene, and co-doped graphene were deposited using CVD from benzene and benzene/pyridine on a nanoporous Ni substrate and tested as the cathode in a Zn–air battery. The synergistic effect of a bicontinuous porous structure and single Ni atomic doping on the nitrogen sites made the co-doped np-graphene-based catalyst achieve ORR and OER performances comparable to commercial Pt/C and IrO_2_. The discharge polarization curves of the co-doped np-graphene-based Zn-air battery revealed a maximum power density of 83.8 mW∙cm^−2^, which is higher than that of an all-solid-state Zn-air battery based on the Pt/C + IrO_2_ catalyst (74.5 mW∙cm^−2^). The co-doped np-graphene-based battery exhibited the highest power density (83.8 mW∙cm^−2^) with the longest testing period (43 h). Those performances were ascribed to the high bifunctional activity, electronic conductivity, and facilitated mass transport provided by the bicontinuous 3D nanotubular graphene network. This structure also presents the advantage of being highly flexible and, thus, could be easily integrated into flexible devices. The bending of the battery at different angles did not affect its performance.

### 4.3. Energy Conversion–Dye-Sensitized Solar Cells

Electrical devices that employ the photovoltaic effect to convert solar energy into electricity, such as dye-sensitized solar cells (DSSC), are a high priority, with solar energy becoming a major component of sustainable energy. In addition to their low production cost, DSSC/organic photovoltaics (OPVs) possess the advantage of being lightweight, transparent, or semi-transparent; easily assembled; and scaled up on flexible substrates. The architecture of a DSSC comprises a photoanode (working electrode–WE), a dye that absorbs the solar energy (sensitizer), an electrolyte (a mediator comprising the redox couple of the liquid DSSC), and a counter electrode (CE, usually a metal electrode).

A free-standing 3D graphene network was employed as both photoanode (WE) and counter electrode (CE) in dye-sensitized solar cells (DSSC), achieving similar, or even higher, performances to a standard DSSC containing TiO_2_ photoanode and Pt counter electrode.

The performance of the main 3D graphene-based DSSCs employing Ru-based light-sensitizing materials, and iodide/triiodide redox couple electrolyte, recorded under standard conditions, is summarized in Table 5.

**Figure 16:**
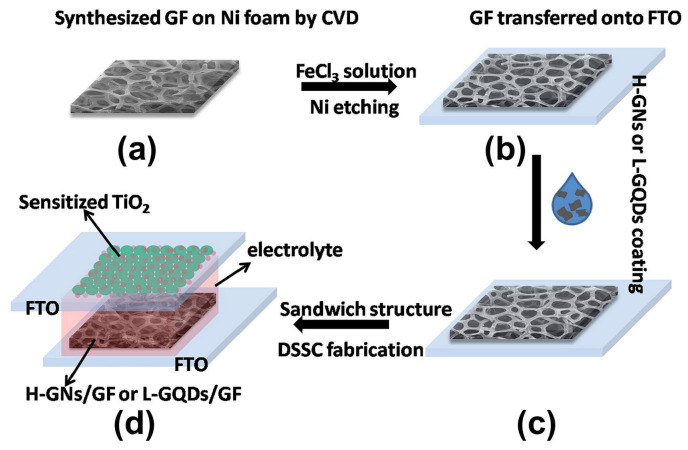
Schematic presentation of a reduced graphene oxide-GF hybrid CE integration in DSSC. (**a**) Template-assisted CVD growth; (**b**) free-standing GF transfer on FTO; (**c**) GF/FTO coating with reduced graphene-based solution (H-GN: heat reduced graphene nanosheets, L-GN: laser reduced graphene nanosheets); (**d**) DSSC configuration. Reprinted from ref. [79] with permission from Elsevier.

In a general approach, in order to be integrated as a CE in the DSSC, the free-standing GF prepared by the conventional CVD method on the Ni foam template with the subsequent nickel etching in the presence of FeCl_3_/HCl is attached to the FTO substrate. A schematic flow diagram of graphene-based CE fabrication and integration into a liquid-state DSSC is presented in Figure 16.

**Figure 17:**
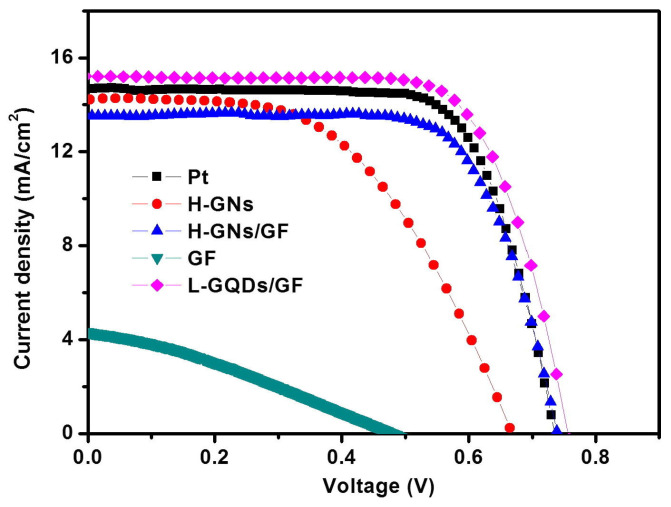
Photocurrent–voltage characteristics of DSSCs with various CEs (Pt, GF, H-GNs, H-GNs/GF, and L-GQDs/GF), measured under AM 1.5 simulated solar illumination. Reprinted from ref. [79], with permission from Elsevier.

Tested under standard conditions, the solar cells based on 3D graphene alone showed marginal performances, with an efficiency of 0.68%, reflected in the low FF and J_SC_ (Figure 17 green line) [79]. The reduced performance of the DSSC solely using 3D graphene as a CE was assigned to either a reduced number of the electrocatalytic active sites in graphene, responsible for the I_3_^−^/I^−^ redox reaction; or to the reduced graphene wettability, responsible for favorable interactions at the electrode–electrolyte interface. To circumvent the reduced number of electrocatalytic active sites present in monolithic 3D graphene networks, both reduced graphene oxide [80] and graphene nanosheets [79] have been used to modify the surface of 3D graphene networks.

When using laser-reduced graphene oxide-3D graphene nanosheets (L-GQD/GF) hybrids [79], the efficiency increased by an order of magnitude to 7.7%, which is comparable to that of a conventional Pt-based DSSC (7.68%) (Figure 17). Among the two types of graphene-based materials, heat-reduced (H-GN) and laser-reduced (L-GN) graphene nanosheets, used to generate 3D graphene nanosheets-graphene foam hybrids, the L-GQD/GF has the highest surface area and catalytic activity (Table 5). The increases are reflected in improved solar cell performance, which is superior to that of H-GN/GF and similar to the conventional DSSC (Figure 17).

A significant increase in the photocurrent density was reported when assembling the CE based on reduced graphene oxide (RGO) and 3D graphene networks (3DGNs), with the RGO-TiO_2_ photoanode in a DSSC [80]; with an overall efficiency of 9.79%, the performance was comparable to that of the conventional cell using Pt as a CE [78]. The high value of the FF, which was 10% higher than that of the RGO and 3D graphene alone, has been ascribed to the low resistance resulting from using the RGO-3DGN as CE.

Besides the classical template-assisted CVD method, three-dimensional nanofoams of few-layer graphene (3D-NFG) were synthesized using pyrolysis of polymer/nickel films under a hydrogen environment, to replace the Pt counter electrode of the DSSC [35,81]. Carbonized carbon and the nickel nano-frame formed were used as the solid carbon source and the catalyst for the growth of graphene under CVD conditions. This took advantage of the expected foam’s large surface area and high conductivity, to test the material as an alternative to the Pt counter electrode of the DSSC. Reported results of 710 mV (V_OC_), 12.2 mA (I_SC_), 60% (FF), and efficiency of 5.2 were similar to those using Pt as a counter electrode [35]. An enhancement in the performance of the solar cell was observed when 3D-NFGs were p-doped with nitrogen by immersion in HNO_3_ [81]. The best performance of 8.46% efficiency, V_OC_ = 713 mV, J_SC_ = 17.2 mA·cm^−2^, and FF = 69% was obtained for the highest doping level (0.5 wt% nitrogen). 

In regards to 3D graphene networks (3DGNs)-based photoanodes, free-standing graphene networks were not directly integrated into the final DSSC, but rather a solution processed with the TiO_2_ (P25) [82] or RGO and TiO_2_ (P25) [83]. Thereafter, the paste was deposited on the conductive glass and heated to 300 °C under an Ar atmosphere, to form the porous photoanode. The best performance for the 3DGN-TiO_2_ photoanode was observed for 1 wt.% loading of 3DGN; with an efficiency of 6.58%, Jsc = 15.4 mA·cm^−2^; Voc = 673 mV; FF = 63.5%, concluding that the thinner the photoanode, the higher efficiency. A slight decrease was observed when increasing the loading to 2 wt.%, due to the small decrease of the Jsc. No further improvement was observed when the counter electrode was also 3DGN. The efficiency, in this case, when both photoanode and counter electrode were 3DGN, was 5.74%, even lower than in the case of 2 wt.% 3DGN (6.01% efficiency) [82].

**Figure 18:**
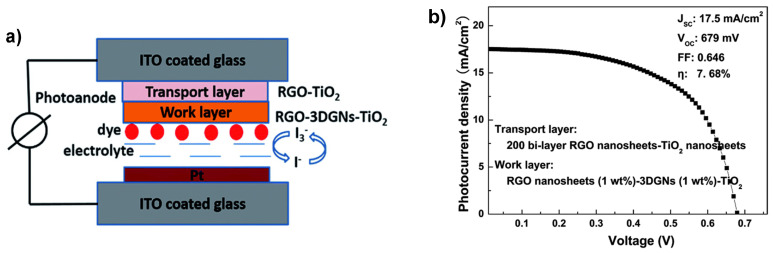
(**a**) DSSC structure; (**b**) J-V characteristic of the device assembled in an optimized manner under standard illumination conditions. Reprinted with permission from ref. [83]. Copyright 2017 The Royal Society of Chemistry.

A somewhat better efficiency was observed when using a reduced-graphene oxide-based transport layer and RGO-3DGN-TiO_2_ photoanode to develop a device (Figure 18a). A good contact between the conducting glass and photoanode, with a reduced dark current, was achieved by intercalating an RGO-TiO_2_ transport layer between the TCO and the 3DGN-RGO-TiO_2_ photoanode. At an optimum number of RGO-TiO_2_ transport layers and with an appropriate degree of reduction, the conversion efficiency reached 7.08%, with an open-circuit voltage and short circuit current density of 682 mV and 16.3 mA·cm^−2^, respectively (Figure 18b) [83].

A new perspective for developing metal-free materials for liquid-DSSC photoanodes was proposed by Loeblein et al., in 2017 [84]. Oxidized-three-dimensional graphene (o-3D-C), with a bandgap of 0.2 eV and a suitable electronic band structure was proven to be a great alternative to the current TiO_2_-based photoanodes. The functional groups on the graphene surface, which promote a reduction in the bandgap and allow an increased dye loading in a very short time, as well as an efficient charge transfer, were introduced by cooling the as grown 3D-C in an air atmosphere. With a large amount of dye adsorption (estimated to be ~450 mg·g^−1^) and a type II alignment structure between o-3D-C and N719 dye (Ru-based sensitizer), these o-3D-C are shortly going to be a component of a metal-free DSSC.

### 4.4. Energy Conversion–Photodetectors

Photodetectors (PD), similarly to solar cells, are devices that convert the incoming photon energy into an electrical signal. According to their application, they require a fast response time, low noise, high dynamic range, and high detection efficiency. Simultaneous detection of multiple wavelengths is possible in an increased sensitivity PD, with a wide field of applicability. Interest in the broadband graphene-based PD was triggered by the seminal work of Dawlaty et al. [85], who show wavelength-independent absorption of single-layer graphene across a very wide energy range (from Vis to THz). As in a PD, the spectral bandwidth is typically limited by material absorption and the response time is governed by carrier mobility, graphene is the ideal material for wide and high-speed photodetection. However, employing 3D graphene networks in photodetection devices is still in its early stages.

Table 6 summarizes the performance of photodetectors based on 3D graphene networks.

**Figure 19:**
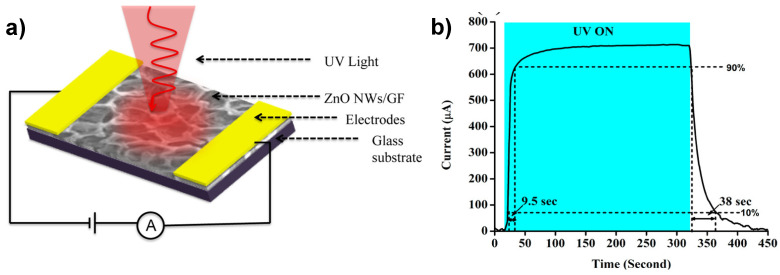
(**a**) Graphic representation of the Zn NW/GF device and (**b**) photo-response of the device at a constant bias voltage of 5 V and UV illumination, indicating the response and recovery time. Reprinted with permission from ref. [86]. Copyright 2015 American Chemical Society.

Template-assisted graphene foams decorated with ZnO nanowires produced by resistive thermal evaporation were connected with conducting silver paste to define an active area of 3 × 3 mm^2^ (Figure 19a). The ZnO NW/GF hybrid demonstrated a photoresponse and recovery times of 9.5 s and 38 s, respectively, with an external quantum efficiency as high as 2490.8% at low UV illumination and an applied bias of 5 V (Figure 19b) [86].

Although the following studies did not employ template-assisted CVD-grown graphene, they have clearly proven the broadband photoresponsivity of 3D graphene networks [87,88].

Starting from graphite oxide, Li et al. synthesized 3DGF with an ultra-broad photoresponsivity [87]. Photodetectors (PDs) based on these three-dimensional (3D) graphene foam (GF) photodiodes with asymmetric electrodes (Au-Ti) showed a photoresponse from ultraviolet to microwave, with wavelengths ranging from 10^2^ to 10^6^ nm. Flat and high photoresponsivities were exhibited over a wide range, from 300 to 2200 nm, which was considered consistent with the absorption spectrum. Moreover, the devices exhibited a high photoresponsivity of 10^3^ A·W^−1^, a short response time of 43 ms, and a 3 dB bandwidth of 80 Hz. The maximum photoresponsivity (R) of 10^4^ mA·W^−1^ was obtained at a bias of −0.1 V, with the rise and fall times estimated to be 48 ms and 116 ms. It is worth noting that when the bias was increased to 5 V, the photoresponsivity reached a high level of 2.4 × 10^6^ mA·W^−1^ at 300 nm. The high performance of the devices was attributed to the photothermoelectric (PTE, also known as the Seebeck) effect in 3D GF photodiodes, rather than to photovoltaic or photoconductive effects. 

In a second study, a 3DG-sponge with visible to mid-infrared (MIR) responsivity was prepared by reduction of GO between two glass slides in an autoclave at 180 °C for 12 h [88]. Given the large noise and high dark current of graphene, as well as the low responsivity of 3DG sponge-based photoconductors, Ge et al. suggested a new method to prepare 3DG-sponge for broadband detection.

**Figure 20:**
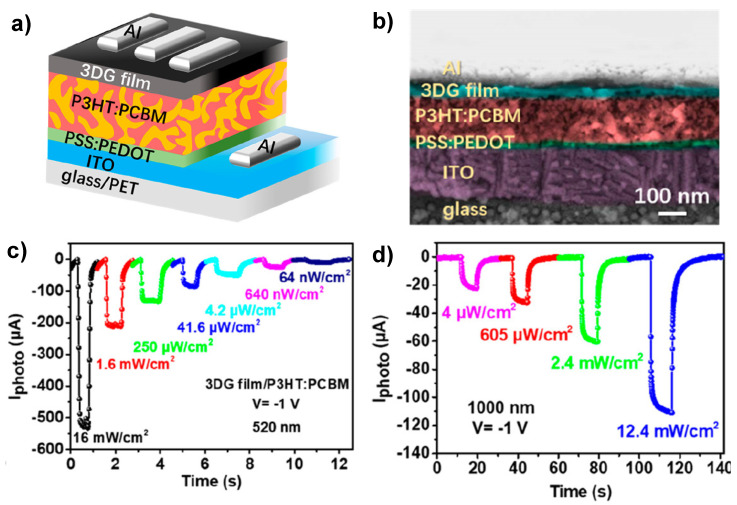
(**a**) Schematic of the 3DG film/organic photodetector; (**b**) Cross-sectional SEM image of the 3DG film/organic photodetector; and temporal photoresponse curves of the 3DG film/organic photodetector under 520 nm (**c**) and 1000 nm (**d**) illumination with different light intensities at −1 V. Reprinted with permission from ref. [88]. Copyright 2022 American Chemical Society.

The appropriate conjunction of materials with efficient near-infrared (NIR) and MIR light absorption and the device’s design for efficient charge separation and transfer allowed them to achieve highly responsive NIR and MIR photodetectors. The developed organic Vis-MIR photodetector used a layered configuration of 3DG film and a classic [6,6]-phenyl C71 butyric acid methyl ester (PCBM)-poly(3-hexylthiophene) (P3HT) bulk heterojunction (BHJ) layer; leading to a device with a total thickness of less than 250 nm. (Figure 20a,b).

The hybrid 3DG/organic photoconductor exhibited the typical photoconductive characteristics, providing high photocurrent gains and responsivity, due to the efficient electron trapping sites present in the PCBM and the functional groups in 3DG. As shown in Figure 20c,d, the device’s response at different light intensities was stable and repeatable. Moreover, the responsivity of the hybrid photodetector in both visible (532 nm) and NIR (1000 nm) ranges increased with the decrease of the incident light, caused by the photocurrent gain in the 3DG film/P3HT: PCBM hybrid system. As noticed, the photocurrent reached 500 µA under 520 nm laser illumination at 16 mW·cm^−2^ and a bias of −1V. The responsivity achieved values of 5.8 × 10^5^ AW^−1^ under 520 nm illumination at 0.4 nW·cm^−2^ irradiance and a bias voltage of −1 V, yielding a photocurrent gain as high as 1.4 × 10^6^. The extended responsivity to the NIR region, mainly ascribed to the graphene sponge absorption, revealed a stable and reliable photoelectric response. Thus, under 1000 nm laser irradiation the device’s responsivity was 0.6 A·W^−1^, with an incident irradiance of 2.4 mW·cm^−2^; reaching a responsivity of 108 A·W^−1^ for an incident power of 4 µW·cm^−2^.

## 5. Conclusions

In summary, the exploration of template-assisted CVD-grown graphene in energy storage and conversion has been highlighted. Although the CVD synthesis of graphene foams seems to have reached its maturity, with the 3D graphene networks being deposited on either metallic or non-metallic substrates from various carbon precursors, the precise control of pore size and porosity is a big challenge and requires attention, as there is a direct relationship between the properties of the graphene-based electrodes and their microstructure. According to the reported studies, most of the 3D graphene electrodes feature randomly distributed pores, with sizes between tens and hundreds of nanometers. 

The properties of 3D graphene networks, especially the high conductivity and specific surface area confer graphene significant potential to function as a binder-free current collector electrode in batteries and supercapacitors. To extend their functionality, considerable efforts have been dedicated to the development of 3D graphene-based hybrids with high active material loading. The process of GF decoration with different active materials allowed, not only the control of mass loading, but also a well-defined morphology of the active materials. Among the tested active materials were metal oxide, sulfides, silicon, and hydroxides of different shapes, from nanoparticles to nanowires, and nanosheets.

Although the specific capacitance and energy density, the rate capability, and cycling life of the hybrid electrodes were superior to those of the active materials alone, they were not at the level of commercially available energy storage devices (supercapacitors 5–8 W h L^−1^, lead-acid 50–90 W h L^−1^ and Li-ion batteries 250–850 Wh L^−1^). There has also been significant progress in their cyclability, which has reached the lower limit of commercial standards for LIBs, between 400 and 1200 cycles, or supercapacitors, which should be not less than 10,000 cycles. For instance, the cyclability of most of the reported supercapacitors is in the range of 500 to 5000 cycles, with a few examples of 10,000 cycles (e.g., ternary composite electrodes based on GF/CNT/MnO_2_, hierarchical mesoporous graphene, and Ni(OH)_2_/G-NG/Ni(OH)_2_). On the other hand, the cycling ability of the reported LIBs was in the range of 300 to 500 cycles. 

With regard to solar cells and photodetection applications, they are at an incipient stage, with the best performances approaching those of standard DSSC containing a TiO_2_ photoanode and Pt counter electrode.

## Figures and Tables

**Table 1:** The statistical data of published articles on the indicated topics over the 2011−2022 time period.

Topics	Results from Web of Science	Articles	Review Articles	Open Access	Peak Year
3D graphene foam + chemical vapor deposition	298	283	15	47	2018 (46)
3D graphene foam + chemical vapor deposition + energy storage	97	89	8	16	2016 (19)
3D graphene foam + chemical vapor deposition + energy conversion	23	21	2	4	2016 (7)
3D graphene foam + chemical vapor deposition + energy storage + energy conversion	20	18	2	4	2016 (6)
3D graphene foam + chemical vapor deposition + supercapacitors	76	68	8	13	2016 (14)
3D graphene foam + chemical vapor deposition + batteries	63	55	8	10	2016 (15)
3D graphene foam + chemical vapor deposition + supercapacitors + batteries	14	11	3	2	2017 (4)

**Table 2:** The main properties of 3D graphene foams synthesized by CVD.

3D Graphene Macrostructures	SSA (m^2^·g^−1^)	Density (mg·cm^−3^)	Number of Layers	Pore Size	Electrical/Thermal Conductivity (S·cm^−1^/Wm^−1^K^−1^)	Mechanical Property	Ref.
Graphene foam (GF)	~850	~5	~3 average	-	~10 S·cm^−1^ (GF/PDMS composite)	Flexible (GF/PDMS composite)	[4]
3D graphene foam	-	~3	1–3	~300 nm (average)	~30 Ω	No significant electrical resistance change over 200 mechanical bending cycles	[14]
3D graphene network	500–600	-	~3	-	~600 S·cm^−1^ (1.6 Ω·sq^−1^)	-	[16]
3D graphene films	-	-	7–11 or 6–12	1–4 μm	11.6 S·cm^−1^ (3D-GF-1000/PDMS)	Stretching the sample without any apparent cracks observed	[19]
Graphene foams	-	-	1-few-multilayer	~1 μm	~20 Ω (equivalent series resistance)	-	[20]
Solvent-free 3D graphene network	-	~50–100	1–4	10–20 μm	~1600 S·cm^−1^	Strong enough to avoid collapsing without the copper skeletons support	[21]
Ultrathin graphite foam and hybrid foams	1.61 (UGF) 1.19–24.2 (hybrid foams)	19 (UGF) 48–188 (hybrid foams)	-	-	2.4 Wm^−1^K^−1^ (UGF) 2.6–16.5 Wm^−1^K^−1^ (effective composite thermal conductivity)	-	[22]
3D micron-porous graphene foam	316	~28	7–10	-	451 S·cm^−1^	-	[24]
Hierarchical graphene foam	-	-	1–13	several hundreds of micrometers	2.28 Wm^−1^K^−1^ (thermal conductivity of HGF in paraffin wax)	-	[26]
High-density three-dimension graphene macroscopic objects (3D-GMOs)	~560	~22	1–10	1–50 μm	~12 S·cm^−1^	-	[28]
3D graphene foams	145	73	>5	Micron scale pores	17.5 S·cm^−1^	Flexible covered with PDMS	[29]
3D graphene foam	~850	~5 (corresponding to 99.7%)	1-few	-	~10 S·cm^−1^	Mechanically robust and flexible	[52]
3D graphene networks	500–600	4.8–5.5	≤3	60–130 µm	550–600 S·cm^−1^	-	[59]
3D tubular graphene network	970	1.6–153 (9.8)	2–3	-	-	Young’s modulus 1.74 MPa at ρ = 9.8 mg·cm^−^^3^	[60]
3D nanoporous graphene monolith	1591	40–60	1–2	∼2.62 cm^3^·g^−1^ pore volume	25.2/32.5 S·cm^−1^	Yield stress and modulus >0.15 and 9.5 MPa	[61]

**Table 3:** Summary of the main performance features of several electrode/supercapacitor configurations using 3D graphene-based electrodes.

Electrode Configuration	Specific Capacitance (Electrode/Supercapacitor)	Energy Density (Max)	Power Density (Max)	Ref.
flexible 3D graphene networks, loaded with MnO_2_	130 Fg^−1^, calculated for the entire electrode; 465 Fg^−1^ for the sample with mass loading of 0.1 mg·cm^−2^ at a scan rate of 2 mVs^−1^;	6.8 Whkg^−1^	62 Wkg^−1^	[64]
graphene/carbon nanotubes/Mn_2_O_3_; (graphene/CNT/Mn_2_O_3_)	~370 Fg^−1^ at 100 mAg^−1^; ~180 Fg^−1^ after ~1000 cycles of charge/discharge;			[46]
flower-like NiCo_2_O_4_ fabricated on 3D graphene foam	1402 Fg^−1^ at the current density of 1 Ag^−1^			[36]
3Dgraphene/Ni_3_S_2_	11.529 F·cm^−2^ at 2 mA·cm^−2^; 10.424 F·cm^−2^ at 5 mA·cm^−2^; 9.448 F·cm^−2^ at 10 mA·cm^−2^; 9.12 F·cm^−2^ at 20 mA·cm^−2^;			[65]
3D foam-like MnO_2_ film/multilayer graphene film/Ni foam	714 mF·cm^−2^ (14.28 F·cm^−3^);	0.634 mWh·cm^−3^	126 mW·cm^−3^	[37]
3D vertically aligned Ag nanoplates on nickel foam-graphene (NFG)	900 Fg^−1^ at an applied current density of 0.5 Ag^−1^;	35 Whkg^−1^ at a power density of 0.91 kWkg^−1^; 26 Whkg^−1^ at a power density of 12.4 kWkg^−1^;		[66]
nitrogen-doped graphene foam/CNT/MnO_2_	284 Fg^−1^ at the scan rate of 2 mVs^−1^;			[67]
graphene on both Ni and Ni-Cu foams	289.7 Fg^−1^ at the current density of 1 Ag^−1^;			[18]
3DG/ZnO	554.23 Fg^−1^ at 5 mVs^−1^	650 Ahg^−1^		[47]
3D GF with small pore size	100 Fg^−1^	11.2 Whkg^−1^	10.1 kWkg^−1^	[20]
NiCo_2_O_4_-MnO_2_/ graphene foam	2577 Fg^−1^ at the current density of 1 Ag^−1^	55.1 Whkg^−1^	187.5 Wkg^−1^	[68]
Ni (OH)_2_/G-NF/Ni (OH)_2_	991 Cg^−1^ at a current density of 1 Ag^−1^	49.5 Whkg^−1^ 7500 Whkg^−1^	750 Wkg^−1^ 26.9 Wkg^−1^	[69]
hierarchical nanoporous graphene (hnp-G)	38.2 F·cm^−3^	2.65 mWh·cm^−3^	20.8 W·cm^−3^	[70]
NiO on 3D graphene	~816 Fg^−1^			[5]
nickel foam coated with graphene Ni(OH)_2_/graphene	1440 Fg^−1^ at charge/discharge current density of 10 Ag^−1^	current density of 20 Ag^−1^		[38]
close-packed hollow 3D graphitic bicontinuous foam	~20–30 µF·cm^−2^; 1 mF·cm^−2^ was achieved for a 10-layer sample of 0.5 µm diameter spheres	382 mJ·cm^−3^	3.2 W·cm^−3^	[30]

**Table 4:** The performance of several battery configurations where 3D graphene was used.

Graphene-Based Materials	Capacity	Ref.
**Lithium-Ion Batteries (LIB)**		
MoO_2_ nanoparticles grown on 3D-graphene	975.4 mAhg^−1^ at the 50 mAg^−1^ current density; 537.3 mAhg^−1^ at the 1000 mAg^−1^ current density;	[44]
WS_2_-3D graphene	927 mAhg^−1^ at the 100 mAg^−1^ current density; 416 mAhg^−1^ at the 1500 mAg^−1^ current density;	[48]
MnO_2_ nanoflakes assembled on GF	1200 mAhg^−1^ at the 500 mAg^−1^ current density; ~500 mAhg^−1^ at the 5 Ag^−1^ current density;	[55]
GF/SiNWs composite	2949 mAhg^−1^ at the 200 mAg^−1^ current density;	[72]
NiMoO_4_ (NWAs)/3DGF composites	1088.02 mAhg^−1^ at the 200 mAg^−1^ current density; 867.86 mAhg^−1^ after 150 cycles	[56]
3D graphene/LiFePO_4_ composite	109 mAhg^−1^ at 10C 158 mAhg^−1^ at 0.2C	[16]
mesoporous nanostructure Fe_3_O_4_ on 3D graphene	780 mAhg^−1^ at 1C and 350 mAhg^−1^ at 10C up to 500 cycles;	[73]
**Lithium-Sulfur Batteries (LSB)**		
3D Li_2_S/graphene hierarchical architecture	894.7 mAhg^−1^ at 0.1C; (1C corresponds to a current density value of 1166 mAg^−1^);	[74]
sulphur-FLG foam three-dimensional cathode	high-rate discharge capacity retention for up to 400 discharge/charge cycles at a high current density of 3200 mAg^−1^	[75]
3D graphene with mesopores	1187 mAhg^−1^ at 1C; (1C = 1672 mAg^−1^, based on sulfur) 852 mAhg^−1^ after 500 cycles	[76]
metal-free sulfur cathode using 3D-MPGF as a current collector	1187 mAhg^−1^ at 0.1C and 618 mAhg^−1^ at 10C up to 50 cycles;	[24]
**Zn-air Batteries**		
codoped np-graphene-based Zn-air battery	power density of 83.8 mW·cm^−2^	[77]

**Table 5:** Performance of the 3D graphene-based DSSCs.

Electrode Materials	Jsc (Short Circuit Current Density)	Voc (Open Circuit Voltage)	FF (Fill Factor)	Overall Efficiency	Observations	Ref.
Standard TiO_2_/Pt commercial	18.2 mA·cm^−2^ 5.09 mA	720 mV 691 mV	73 61	10 5.93		[78] Solaronix
CE: L-GQD/GF CE: H-GN/GF	15.21 mA·cm^−2^ 13.52 mA·cm^−2^	760 mV 740 mV	72 70	7.7 7.1	CE’s SSA: ~422 m^2^g^−1^ CE’s SSA: ~330 m^2^g^−1^	[79]
CE: RGO-3DGN WE: RGO-TiO_2_	20.6 mA·cm^−2^	700 mV	65.2	9.79		[80]
CE: p-doped 3D-NFG	17.2 mA·cm^−2^	713	69	8.46	3D-NFG from PVA-NiCl_2 ×_ 6H_2_O films, and nitrogen doping in HNO_3_	[81]
CE: 3D-NFG	12.2 mA	710 mV	60	5.2	3D-NFG from PVA-NiCl_2 ×_ 6H_2_O films	[35]
CE: Pt WE: 1wt% 3DGN -P25 composite	15.4 mA·cm^−2^	673 mV	63.5	6.58%	The thinner the WE, the higher the efficiency	[82]
WE:3DGN-RGO-TiO_2_	16.3 mA·cm^−2^	682 mV	63.7	7.08	1% RGO	[83]
Oxidized three–dimensional graphene (o-3D-GN)	-	-	-	-	A stand-alone, TiO_2_-free photoanode for DSSC.	[84]

**Table 6:** The performance of photodetectors based on 3D graphene networks.

PD Architecture	External Quantum Efficiency/Responsivity	Illumination Power and Applied Bias	λ (Wavelength)	Response Time Rise/Recovery	Ref.
ZnO NW on GF	2490.8%	1.3 mW·cm^−2^ @ 5 V	UV	9.5/38 s	[86]
3DGF photodiode with Au-Ti electrodes	0.2–0.02 mA·W^−1^ 10^4^ A·W^−1^–maximum 2.4 × 10^6^ mA·W^−1^	0.05 V −0.1 V 5 V	UV to µW (405 nm−1.36 mm) 532 nm 300 nm	40 ms 48/116 ms	[87]
Photoconductive characteristics of GO sponge in the organic PD based on the classical P3HT-PCBM BHJ configuration			Vis to MIR	24/114 ms 1.04/2.52 s	[88]
	5.8 × 10^5^ A·W^−1^	0.4 nW·cm^−2^ @ −1 V	520 nm		
	6.25 × 10^3^ A·W^−1^	10 nW·cm^−2^ @ −1 V	520 nm		
	2 × 10^4^ A·W^−1^	1 nW·cm^−2^ @ −1 V	520 nm		
	Similar R	-	450 and 635 nm		
	0.6 A·W^−1^	2.4 mW·cm^−2^	1000 nm		
	108 A·W^−1^	4 µW·cm^−2^	1000 nm		
	Clear and stable R	700 mW·cm^−2^	10.6 µm		

## Data Availability

Not applicable.
